# Monitoring of Executive Functions During Awake Glioma Surgery: A Standardized Multicenter Protocol

**DOI:** 10.1227/neuprac.0000000000000152

**Published:** 2025-08-08

**Authors:** Maud J. F. Landers, Bart Brouwers, Anne M. Weggelaar, Eva van Breugel, Wouter De Baene, Tessa Meijerink, Martine Wilbers, Pierre A. Robe, Martine J. E. van Zandvoort, Eelke M. Bos, Djaina Satoer, Arnaud P. J. E. Vincent, Isabelle Poisson, Marion Barberis, Emmanuel Mandonnet, Geert-Jan M. Rutten

**Affiliations:** * Department of Neurosurgery, Elisabeth-Tweesteden Hospital Tilburg, The Netherlands;; ‡ Tranzo, Scientific Centre for Care and Wellbeing, Tilburg University, Tilburg, The Netherlands;; § Department of Cognitive Neuropsychology, Tilburg University, Tilburg, The Netherlands;; ‖ Department of Neurology and Neurosurgery, University Medical Centre Utrecht, Utrecht, The Netherlands;; ¶ Department of Neurosurgery, Brain Tumor Center, Erasmus MC Cancer Institute, Erasmus University Medical Center, Rotterdam, The Netherlands;; # Service of Neurosurgery, Lariboisière Hospital, Paris, France;; ** University of Paris, Paris, France

**Keywords:** Executive functions, Awake neurosurgery

## Abstract

**BACKGROUND AND OBJECTIVES:**

Currently, there are no standardized clinical mapping protocols for monitoring of executive functions during awake glioma surgery, primarily due to a lack of evidence-based data for cognitive mapping. By aligning procedures and documentation practices across institutions, clinicians can overcome the current fragmentation in the field and iteratively work toward generating reproducible, high-quality Data sets that will better clarify the clinical relevance of white matter pathways involved in executive functions. A previously conducted pilot study led to the development of a standardized monitoring protocol and demonstrated that pooling of data is feasible when surgical teams commit to the study requirements. The primary goal of this multicenter study protocol is to investigate whether using this standardized protocol can identify white matter tracts involved in executive functions.

**METHODS:**

In this prospective, clinical observational study, we will continue data collection in 4 neurosurgical departments from the previously conducted pilot study and expand to other hospitals providing neurosurgical care. We aim to include adult patients that will undergo awake primary glioma surgery and undergo monitoring of executive functions with a uniform set of tasks for the following white matter tracts: frontal aslant tract, superior longitudinal fasciculus II and II, arcuate fasciculus, inferior fronto-occipital fasciculus. Data will be collected in a standardized manner for each patient before, during, and after surgery.

**EXPECTED OUTCOMES:**

The primary objective of this study was to determine if executive functions can be effectively monitored using a standardized protocol during awake glioma surgery in multiple neurosurgical centers.

**DISCUSSION:**

Despite limitations inherent to multicenter and observational studies, this study represents a necessary step toward developing a validated uniform way of collecting intraoperative findings on mapping of executive functions. The generation of high-quality Data sets is highly needed to extend the scientific basis for monitoring of white matter pathways involved in executive functions.

**ABBREVIATIONS:**
AF: arcuate fasciculus
DES: direct electrical stimulation
DWI: diffusion-weighted imaging
SLF: superior longitudinal fasciculus.

## RATIONALE AND BACKGROUND INFORMATION

Over the past few decades, the connectomic architecture of the brain has been increasingly acknowledged in neurosurgical procedures. However, its exact functional role needs further elucidation. A method to directly study the functionality of anatomic structures is direct electrical stimulation (DES) during awake neurosurgery. DES induces a transient disturbance of the underlying neural tissue.^1,2^ This tissue is considered to be clinically relevant (ie, ‘indispensable’ or ‘eloquent’) for a particular function if DES results in a deficit on a task that intends to measure the particular function. Hence, DES of white matter offers a unique opportunity to investigate the functional contribution of brain connections.^3^ In clinical practice, DES is widely used by neurosurgeons and is currently the gold standard for identifying functional areas during neurosurgical procedures. In the past, DES was only used for identification of cortical areas, mainly involved in motor and language function.^4-7^ More recently, it has become an established tool to identify white matter pathways that are important for sensorimotor, language, and visual functions.^8^ For mapping of higher cognitive functions other than language, such as executive functions, there are no clinical mapping protocols yet. Cognitive tasks are generally selected in a patient-specific and site-specific manner based on symptoms, tumor location, and preference of the involved surgeon or neuropsychologist.^9^

Few studies addressed DES of white matter tracts involved in executive functions. In 2017, a general survey was sent to 28 members of the European Low Grade Glioma Network to establish the current practice on, among others, intraoperative assessment of cognitive functions.^10^ Their results demonstrated that the only commonly used task was picture naming, whereas tasks for other cognitive functions (executive functions, attention, visuospatial awareness) and emotion tasks were sparsely used. This is in accordance with 2 recent reviews that described language functions to be widely monitored during awake brain surgery, whereas other cognitive functions are not.^8,11^

Recently a systematic review was performed on the involvement of white matter pathways in executive functions and spatial cognition (specifically: inhibitory control, working memory, cognitive flexibility, and visuospatial awareness).^12^ Only 12 studies explicitly clarified which white matter structures were stimulated during electrical stimulation, and the used methods and tasks were heterogeneous. These results are clearly insufficient to unequivocally establish intraoperative relationships between white matter pathways and executive functions.^13^ In addition, the heterogeneity of study types and used methods highlights the need for the development of a standardized protocol for intraoperative mapping of executive functions. Because there are currently no evidence-based DES data for cognitive mapping, this raises a chicken-and-egg problem, because such a protocol for executive functions can initially only be based on consensus and expert knowledge.^13,14^ A standardized protocol, even if initially based on expert opinion rather than scientific evidence, would provide a starting point for accumulating structured data across centers. By aligning procedures and documentation practices across institutions, clinicians can overcome the current fragmentation in the field and in an iterative manner work toward generating reproducible, high-quality Data sets that will better clarify the white matter pathways involved in executive functions.

As a first step, we previously conducted a pilot study to explore current cognitive monitoring practice in 4 international expert centers and assess the feasibility of developing such a standardized protocol and pooling data.

### Summary of Results of the Pilot Study

The 4 hospitals all used different tasks to monitor executive functions.^15^ Only the picture naming task for language was used on all settings. Initially, an expert consensus panel was convened to agree on (1) a set of intraoperative tasks and (2) standardized methods for uniformly collecting and documenting findings during intraoperative monitoring. This consensus panel served as a foundation to explore current practices and establish a baseline for the start of the current observational multicenter study. Based on the consensus, we outlined study requirements for uniform tasks to monitor executive functions and ensured their consistent administration across hospitals.

The pilot study has been conducted with 5 patients in each of 4 neurosurgical centers (ie, Tilburg, Utrecht, Paris, Rotterdam) to check whether surgical teams could fulfil the study requirements as outlined by the consensus panel. After operating a total of 20 patients, we conducted evaluation meetings per hospital, in which we discussed the current state-of-the-art and feasibility of the different tasks as agreed upon (Table 1).^15^ These results were combined with the findings from the systematic review^12^ and retrospective studies^15^ and resulted in an overview of white matter tracts and recommendations for monitoring (Table 2). Tasks for language and visuospatial function were also included as they are also commonly monitored tasks for several of the white matter tracts involved in executive functions.

**TABLE 1.** Set of Tasks That Were Adapted for Intraoperative Use^15^

Intraoperative test	Domains cognitive	Type of errors	Description
**Executive functions**			
Stroop interference	Inhibition	Error Slowing	2 lines of 6 color words are presented on the screen in different colors. The color of the printed ink has to be named
Stroop with shifting	Cognitive flexibility	Not shifting Error Slowing	When a word is surrounded by a square the word should be read instead of naming the color
Digit span forward	Verbal short-term memory (recall)	Item error Order error Missing item	Repeat sequence of numbers that are read out (baseline −1, max 7)
Digit span backward	Working memory	Item error Order error Missing item	Repeat sequence of numbers that are read out in reverse order (baseline −1, max. 5)
Corsi blocks tap test forward	Visual short-term memory	Item error Order error Missing item	Repeat sequence of spatially separated blocks by tapping
Corsi block tap test backward	Visual working memory	Item error Order error Missing item	Repeat sequence of spatially separated blocks in reverse order
Dual task	Attentional capacity/shifting	Stop moving arm Motion-programming disorder Stop counting	Moving contralateral arm while counting or naming
TMT-B	Cognitive flexibility Inhibition	Shifting error Sequence error Slowing	Drawing lines to connect the circles in an ascending pattern, but with the added task of alternating between the numbers and letters (1-A-2-B-3-C-4-D-5-E or reverse A-1-B-2-C-3-4-D-5-E)
**Language and speech**			
Picture naming	Word retrieval Speech	Speech arrest Anomia Dysarthria Speech apraxia Semantic or phonemic paraphasia Slowing	Picture is presented on the screen and has to be named “This is …”. (only the pictures that were correctly recognized at baseline are presented)
Odd-picture-out	Semantic judgment and word retrieval Speech	Error, the odd picture out is not (verbally) identified or the wrong picture is (verbally) identified Slowing	Three pictures are presented on the screen and the semantically odd one out has to be named. (only the pictures that were correct at baseline are presented)
Repetition			
Words Nonwords sentences	Verbal short-term memory (recall) Fluency Speech	Nonfluent Dysarthria Phonemic or semantic paraphasia Jargon Missing words	Auditory sentence processing and repetition
Sentence completion			
Semantically induced Nonsemantically induced	Semantic judgment Word retrieval Fluency Speech	Nonfluent No response Incorrect grammar Incorrect semantics Dysarthria Paraphasia	Completion of an unfinished sentence by filling in a plausible missing word or phrase
**Visuospatial**			
Line bisection task	Visuospatial awareness	Right or left lateralized placement of line	Place a vertical line through the center of a series of horizontal lines

It is important to note that these tasks are not used as a diagnostic tool to measure neuropsychological impairments, but that they are used as a tool to instantly identify areas that are potentially involved in a particular function. TMT-B, Trail Making Test part B.

**TABLE 2.** Overview of White Matter Tracts and Recommendations for Monitoring^12^

White matter pathway	Network	Hemisphere	Function	Proposed task
Frontal Aslant Tract (FAT)	Fronto-opercular network	Dominant and nondominant	Speech and motor initiation	Picture naming Dual task Sentence completion
			Working memory	Digit span and Corsi blocks
			Inhibition	Stroop
			Cognitive flexibility	TMT-B (or Stroop with shifting)
Superior Longitudinal Fasciculus II (SLF II)	Frontoparietal network	Dominant	Working memory	Digit span
	Dorsal language network	Dominant	Speech Language	Counting Picture naming
		Nondominant	Visuospatial awareness	Line bisection task
			Visual working memory	Corsi blocks backward
Superior Longitudinal Fasciculus III (SLF III)	Frontoparietal network	Dominant	Phono-sensory-motor integration Short-term maintenance (phonological loop)	Repeat sentences Counting Digit span forward
		Nondominant	Short-term maintenance (visual)	Corsi blocks forward
Arcuate Fasciculus (AF)	Dorsal language network	Dominant	Repetition Word retrieval	Repeat words, nonwords, sentences Picture naming Odd-picture-out
Inferior Fronto Occipital Fasciculus (IFOF)	Ventral language network	Dominant and nondominant	Semantics	Picture naming Odd-picture-out
			Inhibition	Stroop

The second pilot study requirement was to acquire uniform and standardized data from intraoperative monitoring so that neuroimaging data including a preoperative MRI-diffusion-weighted imaging (DWI) scan (for tractography), recorded intraoperative stimulation sites, and patient performance (ie, types of errors) can be used for post hoc analyses. During the evaluation meetings, the value of documenting positive stimulation sites and documenting the type of response was acknowledged. Especially relevant was to determine which white matter tract was most likely stimulated using DES and resulted in which type of response, ie, correct or type of incorrect response. The error types we agreed upon to register are presented in Table 1 column 3. Furthermore, the value of documenting negative response sites during stimulation was also discussed, and we agreed that within clinical routine collection of negative task performance is best registered at positive sites for other tasks.^15^ This information is still valuable as it will teach us at which white matter location, with which task intraoperative anatomofunctional correlations can be detected.

The pilot study resulted in a standardized protocol and demonstrated that pooling of data is feasible when surgical teams commit to the study requirements. To extend the scientific basis for monitoring of executive functions, we will need to increase the sample size by setting up a larger multicenter observational study.

## STUDY GOALS AND OBJECTIVES

The primary goal of the current study was to investigate whether intraoperative electrical stimulation can identify white matter tracts involved in executive functions. To achieve this, we need to expand the protocol to other Dutch hospitals providing neurosurgical care to make sure that they use a uniform set of tasks to monitor executive functions using DES during awake brain tumor surgery. Second, we need to ensure a consistent method of documenting this.

This is a clinical observational study in which surgical teams continue to work according to their usual clinical best practices. However, when monitoring executive functions intraoperatively, they are asked to follow the study protocol to assess executive functions and register this in a standardized manner.

## STUDY DESIGN

This is a prospective, clinical observational study that will take place in the same 4 neurosurgical hospitals which were included in the pilot study (for hospital names see title page), and will be expanded to other Dutch hospitals providing neurosurgical care.

### Subjects

#### Inclusion Criteria

Adult patients (older than18 years) surgically treated with awake brain surgery for a primary glioma who will undergo executive monitoring with at least one of the uniform tasks (Table 1) for one or more of the following white matter tracts: frontal aslant tract, superior longitudinal fasciculus (SLF) II and SLF III, arcuate fasciculus (AF) or inferior fronto-occipital fasciculus.

#### Exclusion Criteria

- Age below 18 years
- A recent history of other major medical illnesses in the past year before surgery
- Previous craniotomy
- Previous treatment of glioma
- Severe psychiatric or neurological disorders in the past 2 years
- Lack of language skills in native language

## METHODOLOGY

Data will be collected in a standardized manner for each patient before, during, and after surgery according to the agreed upon documentation outcome, translated into a practical checklist and aligned study form per patient (Figure 1).

FIGURE 1.**A** and **B**, Study form and checklist Monitor and Map. At the first page, general information about the included patient can be filled in. At the second page, the results from the cortical and white matter mapping can be filled in per marker, whereby current (milliampere), pulse width (milliseconds), task (from Table 1) and result (detailed description of response of the patient) are written down.

**Figure:**
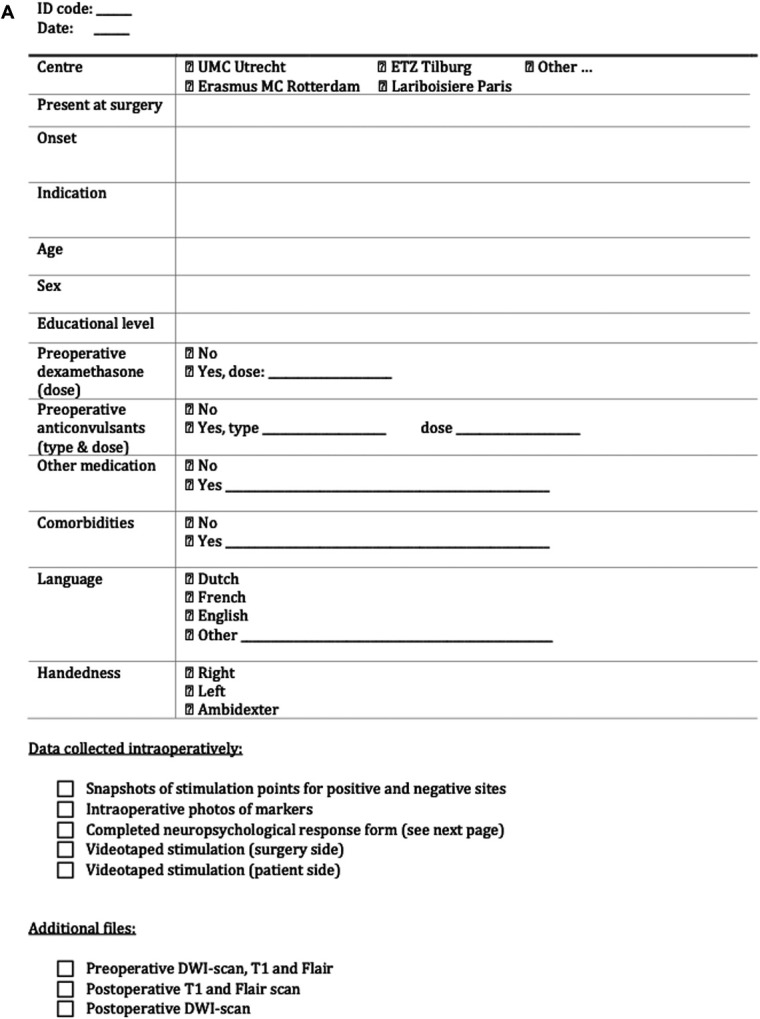

**Figure:**
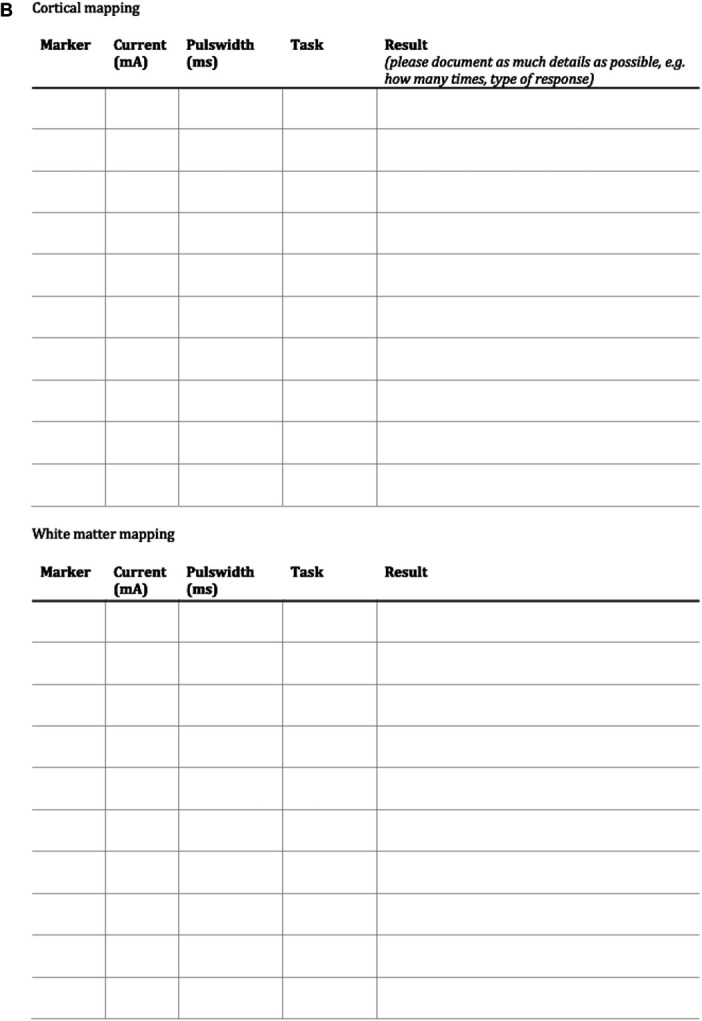

### Before Surgery

Demographic variables (eg, age, sex, educational level) and current patient status will be abstracted from hospital medical records. In addition, presurgical MRI scans including a (3D) *FLAIR* and *T1-contrast* scan and the DWI *bvec, bval*, and NIfTI files will be collected from the radiology department.

### During Surgery

The following intraoperative findings will be collected using the study form: snapshots of the cortical and white matter stimulation points (via the navigation system), intraoperative photographs of the markers and videos on both the surgery side and the patient side (Figure 1) and intraoperative time.

### After Surgery

Postsurgical MRI scans including MRI-DWI scans will be collected from the radiology department, which will be used for among others assessing extent of resection. Pathological examination for tumor type and tumor grade will also be collected.

### Flow Diagram

**FIGURE 2.**
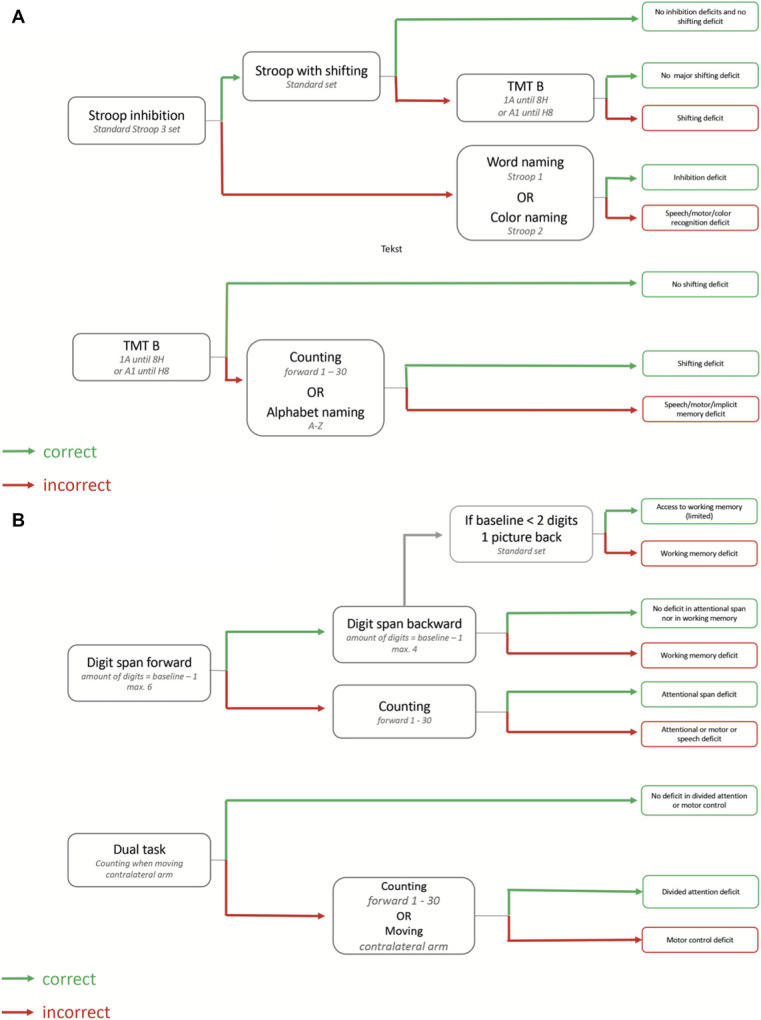
**A** and **B**, Flow diagram for intraoperative monitoring. The flow diagram is modular, meaning it can start from any of the first 4 boxes. The boxes contain the task. If the patient performs the tasks correctly, one follows the green arrow that leads to the next box containing the next task. If the patient performs the tasks incorrectly, one follows the red arrow that leads to the next box containing the next task. The last column of boxes provide an indication about the cognitive domain at risk.

In Figure 2 we present a concept flow diagram for executive monitoring during awake surgery. The flow diagram is based on expert consensus statements of the 4 European neurosurgical centers from the pilot study and was developed as a guideline for executive monitoring of the following cognitive domains: attention, inhibition, working memory, and cognitive flexibility. The flow diagram is modular, meaning it can start from any of the domains and can be adapted to the pre-existing functioning of the patient. In the current study, it will function as a tool to aid in executive monitoring.

### Implementation

The project will start with a group meeting to finalize arrangements regarding the study objectives and methodology presented in this article. Subsequently, a group interview will be conducted at each hospital every 3 months to discuss: (1) the study protocol, (2) intraoperative data collection methods, (3) process flow, and (4) outcomes. These interviews aim to evaluate both intended and unintended study results, refining the methodology and materials as needed. All relevant staff (eg, physicians, research assistants, linguists, and neuropsychologists) will be invited to participate. The meetings will be facilitated by an expert in implementation and change management. All participants will be briefed in advance about the meeting's purpose and structure.

Meetings will be recorded, transcribed verbatim, and supplemented with observational notes. Thematic analysis, following Braun and Clarke's 6-step framework,^16^ will be conducted every 3 months. Findings will be shared with the teams during subsequent group interviews to foster continuous improvement. Collectively, the data will form a Learning History,^17^ providing valuable insights for hospitals seeking to implement the study's outcomes.

## DISCUSSION

Limitations inherent to multicenter and observational studies are present in this study. Even when using a uniform set of tasks, there will be differences between hospitals as the team composition (neurosurgeon, linguist, neuropsychologist, anesthesiologist) and daily practice differ per hospital. Furthermore, the choice to perform the surgery awake depends on the tumor location and differs per neurosurgical team, which might limit the chance to find consistent results and impede the generalizability. In addition, assessment of the accuracy is also impeded by other interfering factors such as variable patient performance in the awake setting, limited time in the awake period, and tiredness of the patient. Furthermore, at many locations the fibers of 2 or more tracts cross, which limits the ability to test the involvement of a particular subcortical tract in a particular function. For instance, if one wants to test the involvement of the left SLF II in working memory, and stimulates at the site where the SLF II crosses with the lateral part of the corticospinal tract, this would probably result in a motor response, making it impossible to test if the SLF II is involved in working memory at that particular location. Despite these limitations, this study still is a necessary step for working toward a validated uniform way of collecting DES findings of white matter pathways. The heterogeneity between hospitals also allows for valuable additional information and for a more comprehensive overview of where positive sites for higher cognitive functions can be detected in the white matter brain.

### Trial Status

The trial has been funded by ZonMW, the Dutch national organization for Health Research and Development (for project number see title page). The trial started at the first of February 2025 and is registered at https://projecten.zonmw.nl/nl.

### Safety Considerations

Assessment of safety is not applicable to the design of this study.

### Follow-Up

This study focusses on intraoperative findings. Follow-up of neuropsychological assessment is not a primary outcome. However, when one or more positive intraoperative stimulation site(s) for a particular task/set of tasks and white matter tract yield consistent results across patients, it is essential to investigate long-term impact of these functions after surgery to assess the clinical relevance of these tracts. This would further clarify the critical neural correlates for executive functions and the relevance of intraoperative mapping. For instance, if anatomofunctional studies demonstrate a correlation between working memory and left SLF II, and a retrospective series with patients who had surgery near the left SLF II without intraoperative monitoring shows that patients suffer from a permanent deficit after surgery, this would provide converging evidence for the awake mapping of this tract during brain tumor surgery. In the long term, we aim to eventually develop an evidence-based intraoperative protocol for white matter mapping of cognitive functions.

### Data Management and Statistical Analysis

The data from the participating neurosurgical centers will be pseudonymized before they are shared with researchers (Tilburg). The pseudonymized data will be stored separately on a protected server system for processing, archiving, and repository. All participating centers have received a Data Sharing Agreement that includes the Terms and Conditions under which the data can be shared following the General Data Protection Regulation, that was signed by the chairperson of the board of directors of each providing and receiving center.

**FIGURE 3.**
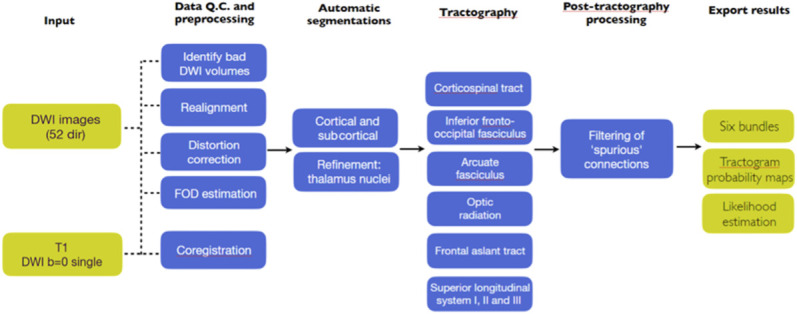
Patient-specific automatic pipeline for automatic tractography. Overview of the automatic pipeline, showing the steps from initial input data, to the output in the form of 8 white matter tracts, derived from Meesters et al.^18^ This automated tractography pipeline will be used to generate the following white matter tracts: inferior fronto-occipital fasciculus, arcuate fasciculus, superior longitudinal fasciculus II and III, frontal aslant tract. The used seed and target regions for each tract are listed in the tractography protocol. FOD, fibre oriented density.

DWI-MRI scans will be processed in the patient-specific automatic pipeline for tractography (Figure 3) on the protected server system and will be used to automatically generate the following white matter tracts: frontal aslant tract, SLF II and SLF III, AF, and inferior fronto-occipital fasciculus. Navigation snapshots and/or DICOM-space coordinates of intraoperative positive response sites are derived from the patients' presurgical DWI-MRI scan to allow for correlating the findings from these sites to the patient's own tractograms. We will calculate whether the sites lay near one or more of the previously mentioned tracts by using a previously developed algorithm (Landers et al 2021, Brain imaging and behavior) that was implemented in Wolfram Mathematica 12 (Wolfram Research, 2019) to calculate the spatial relationship between the stimulation site and the ipsilateral tracts. We will list per white matter tract and per tested function in how many patients positive and negative responses were found by adding up the results from all centers. Please note that, as previously described (see summary pilot study, p. 3), we will only collect data from negative responses at positive sites for other tasks, given that clinical routine does not allow for collection of coordinates from all negative sites. The anonymized data from the group interviews will be stored in the data repository too. Using the FLAIR principles, metadata will be available upon reasonable request for additional studies.

### Quality Assurance

Data quality assurance will be undertaken by cross-monitoring and data quality control in-between hospitals. Monthly meetings will be planned with the data collectors at each site to keep track of the data collection.

### Expected Outcomes

We expect to learn whether the previously named white matter tracts are involved in executive functions and can be monitored with a standardized set of tasks.

### Duration of the Project

We anticipate the data gathering to be complete by the end of 2028 with the data analyses performed by the summer of 2029. Afterward we aim to expand to members of the European Low Grade Glioma Network.

### Project Management

This project is managed by a principal investigator that leads the project. The project group consists of an implementation expert, site coordinators, and coinvestigators, both clinicians (neurosurgeons and neuropsychologists) and researchers.

### Ethics

The Medical Ethics Committee (Medisch Ethische Toetsingscommissie Brabant/22.064) approved the study. An amendment concerning merging and analyzing data collected in the other neurosurgical centers with the data collected in the coordinating hospital was submitted. The Medisch Ethische Toetsingscommissie concluded that the study (including the amendment) met methodological requirements and that there were no medical ethical objections. All patients will be asked for written informed consent to participate and for the use of their data for research purposes.

## COMMENTS

While intraoperative stimulation mapping is the well-established gold standard for performing maximal safe resections of intra-axial tumors, most of the published literature has focused on testing and preserving language and motor functions. However, there is tremendous variability in task selection, stimulation parameters, and data recording which makes evaluation across centers different. Moreover, very few intraoperative assessments focus on executive functions like attention or working memory. The authors here propose a standardization for data collection and recording during stimulation mapping, which is critically important for curating and analyzing big data sets suitable for the application of advanced machine learning/deep learning techniques. They also outline a set of non-motor and non-language tasks and describe a workflow for their use during stimulation mapping. While it remains to be determined how mapping executive functions will impact patient outcomes, extent of resection, and intraoperative times, the efforts described here to standardize and expand our intraoperative testing repertoire are needed to facilitate larger data-driven analyses through collaborative groups with the ultimate goal of improving patient functional and clinical outcomes.

**Jacob Young**

*San Francisco, CA, USA*
